# MMP-9 as a Biomarker for Predicting Hemorrhagic Strokes in Moyamoya Disease

**DOI:** 10.3389/fneur.2021.721118

**Published:** 2021-08-31

**Authors:** Junlin Lu, Jingyi Wang, Zhidong Lin, Guangchao Shi, Rong Wang, Yahui Zhao, Yuanli Zhao, Jizong Zhao

**Affiliations:** ^1^Department of Neurosurgery, Beijing Tiantan Hospital, Capital Medical University, Beijing, China; ^2^Department of Surgical Intensive Care Unit, Beijing Chaoyang Hospital, Beijing, China; ^3^The Second Affiliated Hospital of Chinese Medicine, Guangzhou University of Chinese Medicine, Guangzhou, China; ^4^Department of Neurosurgery, Peking University International Hospital, Beijing, China; ^5^China National Clinical Research Center for Neurological Diseases, Beijing, China; ^6^Stroke Center, Beijing Institute for Brain Disorders, Beijing, China; ^7^Beijing Key Laboratory of Translational Medicine for Cerebrovascular Disease, Beijing, China; ^8^Beijing Translational Engineering Enter for 3D Printer in Clinical Neuroscience, Beijing, China

**Keywords:** blood-brain barrier, hemorrhagic stroke, moyamoya disease, mechanism, MMP-9

## Abstract

**Objective:** This study was conducted in order to investigate the association of matrix metalloproteinase (MMP)-9 levels with phenotypes of moyamoya disease (MMD).

**Methods:** This study included plasma samples from 84 MMD patients. The clinical variables of these patients were reviewed from the medical record. The serum concentration of tight junction, adherens junction proteins, and MMPs (MMP-2 and MMP-9) was determined using the ELISA method. Patients with hemorrhagic-onset MMD were compared with those with ischemic-onset MMD.

**Results:** Compared with pediatric patients, the expression of MMP-9 was significantly higher, while the MMP-2 and vascular endothelial-cadherin were lower in adult patients. In adult subgroup analysis, hemorrhagic MMD patients exhibited significantly higher serum concentrations of MMP-9 compared with ischemic MMD patients. The ROC curve identified that a baseline serum MMP-9 level >1,011 ng/ml may be associated with spontaneous hemorrhage in adult MMD patients with 70.37% sensitivity and 71.88% specificity [area under curve (AUC), 0.73; 95% CI 0.597–0.864; *P* = 0.003]. A late Suzuki stage (>4) (OR 4.565, 95% CI 1.028–20.280, *P* = 0.046) and serum concentrations of MMP-9 >1,011 ng/ml (OR 7.218, 95% CI 1.826–28.533, *P* = 0.005) are risk predictors of hemorrhages in MMD patients. Hemorrhagic-type MMD patients had higher serum levels of MMP-9 and BBB permeability compared with ischemic-type MMD patients. Adult MMD patients had higher serum levels of MMP-9 and BBB permeability compared with pediatric patients.

**Conclusions:** MMP-9 might serve as a biomarker for hemorrhage prediction in MMD. Serum MMP-9 level >1,011 ng/ml is an independent risk factor of MMD hemorrhagic strokes.

## Introduction

Moyamoya disease (MMD) is a chronic cerebrovascular disorder characterized by idiopathic progressive stenosis or occlusion at the terminal portion of the internal carotid artery (1, 2). It has been acknowledged that clinical manifestations and characteristics vary between MMD subtypes (3), including collateral vasculature and blood–brain barrier (BBB) integrity (4, 5). Our previous report also suggested that ischemic and hemorrhagic MMD presented inconsistent angiogenesis potentials (6, 7). However, the underlying pathophysiology leading to MMD phenotypes remains unclear.

Matrix metalloproteinases (MMPs) are calcium-dependent zinc-endopeptidases of the metzincin superfamily which plays an important role in many physiological functions, including extracellular matrix tissue remodeling and immune response (8). They have been recently proposed as possible biomarker in different neurological immune-mediated disorders (9–11). MMP-9 and MMP-2 digest the basal lamina and break down tight junctions, leading to leakage of endothelial barrier and participating in the progress of neoangiogenesis (12). Kang et al. (13) reported increased expression of MMPs in MMD patients compared with healthy controls. A recent study also indicated the involvement of MMP-9 in MMD pathophysiology (14), but whether the expression of MMP-9 contributes to different phenotypes of MMD has not been investigated yet.

The purpose of this study was to investigate the serum levels of MMP-9 and BBB biomarkers among MMD subtypes, hoping to provide evidence of pathophysiological differences underlying MMD phenotypes.

## Materials and Methods

### Subjects and Sample Preparation

We prospectively recruited 84 consecutive patients with MMD at our institution from December 1, 2017, to August 1, 2018. We obtained approval from the institutional research ethics committee (KY2016-048-01) and informed consent from all individual participants and/or the closest relatives. Conventional angiography was applied in all patients to confirm the diagnosis of MMD. The diagnosis of MMD was confirmed with digital subtraction angiography based on the criteria of the Research Committee on Spontaneous Occlusion of the Circle of Willis (2012) (15). Patients with manifestation of intracranial hemorrhage were defined as hemorrhagic type; otherwise, they were defined as ischemic type. The only inclusion criteria for participation was the diagnosis of MMD by means of digital subtraction angiography. To avoid the impact of acute ischemic or hemorrhagic stroke on the serum levels of MMP-9 and BBB biomarkers, we excluded patients who experienced an ischemic or hemorrhagic stroke within 3 months, regardless of the manifestation type (hemorrhagic type or ischemic type). Blood samples were collected before patients underwent the first revascularization surgery.

Blood samples (40 ml) were collected in vacutainers that contain no anticoagulant and sat upright at room temperature for 15–20 min to allow for clotting. Serum was isolated using density-gradient centrifugation over Megafuge 16 (Thermo Scientific, Waltham, MA, USA) for 20 min at 3,000 rpm and stored at −80°C until assayed.

### Data Collection

The clinical characteristics of the patients with MMD obtained at admission were age, sex, body mass index (BMI), initial clinical manifestations (ischemic type and hemorrhagic type), past medical history (including hypertension, diabetes mellitus, hyperlipemia, smoking), neurological status, and imaging findings. BMI was calculated as weight (kg)/height^2^ (m^2^). Suzuki stage was recorded as previously described (16). The posterior circulation involvement was defined as stenosis or occlusion of the posterior cerebral artery with abnormal vessel formation. The decreased regional cerebral blood flow (rCBF) was assessed according to the CT perfusion. Neurological status was recorded using the modified Rankin Scale (mRS) score.

### Biomarker Analyses

The enzyme-linked immunosorbent assay (ELISA) was performed to detect the levels of the MMP-2, MMP-9, occludin (OCLN), junction-associated molecule-1 (JAM-1), claudin 5 (CLDN5), and vascular endothelial-cadherin (VE-cadherin) in serum samples.

MMP-2 and MMP-9 levels in serum samples were analyzed by commercially available ELISA kits (R&D Systems, Minneapolis, MN, USA, MMP200; R&D Systems, DMP900). First, add 100 μl of assay diluent to each well and add 100 μl of standard, control, or sample (20-fold diluted in carbonate buffer) per well and incubate at room temperature for 2 h on a horizontal orbital microplate shaker (0.12-inch orbit) set at 500 rpm. Aspirate each well and wash, repeating the process three times. Add 200 μl of human MMP-2 conjugate/MMP-9 conjugate to each well, cover with a plate sealer, and incubate at room temperature for 1 h on the shaker. Repeat the aspiration/wash. Add 200 μl of substrate solution to each well and incubate for 30 min in the dark on a plate shaker at room temperature. Add 50 μl of stop solution to each well. The color in the wells should change from blue to yellow. Determine the optical density (OD) of each well within 30 min, using a microplate reader set to 450 and 540 nm. Subtract readings at 540 nm from the readings at 450 nm.

JAM-1 levels in serum samples were analyzed by commercially available ELISA kits (Abcam, Cambridge, MA, USA, ab213800). First, add 100 μl standard, control, or sample (4-fold diluted in carbonate buffer) per well and incubate at 37°C for 90 min. Then, add 100 μl biotinylated antibody per well and incubate at 37°C for 60 min. Aspirate each well and wash with 300 μl 0.01 M PBS, repeating the process two times. Add 100 μl ABC working solution and incubate at 37°C for 30 min. Repeat the aspiration/wash. Add 90 μl of prepared TMB and incubate at 37°C in the dark for 20 min. Add 100 μl TMB stop solution and read OD at 450 nm within 30 min.

VE-cadherin levels in serum samples were analyzed by commercially available ELISA kits (Abcam, ab210968). First, add 50 μl standard, control, or sample (200-fold diluted in carbonate buffer) per well. Add 50 μl of the antibody cocktail per well and incubate at room temperature for 1 h on the shaker set to 400 rpm. Aspirate each well and wash with 3 × 350 μl 1 × wash buffer PT. Add 100 μl of TMB substrate per well and incubate for 10 min in the dark on the shaker set to 400 rpm. Add 100 μl of stop solution to each well. Shake plate on a plate shaker for 1 min to mix. Record the OD at 450 nm.

OCLN and CLDN5 levels in serum samples were analyzed by commercially available ELISA kits (LifeSpan BioSciences, Seattle, WA, USA, LS-F21046-1; LifeSpan BioSciences, LS-F21142-1). First, add 100 μl of standard, control, or sample (4-fold diluted in carbonate buffer) per well, cover with a plate sealer, and incubate for 90 min at 37°C. Aspirate the liquid of each well. Add 100 μl of 1 × biotinylated detection antibody per well, cover with a plate sealer, and incubate at 37°C for 1 h. Aspirate each well and wash with 350 μl of wash buffer three times. Add 100 μl of 1 × HRP conjugate working solution per well, cover with a plate sealer, and incubate at 37°C for 30 min. Repeat the aspiration/wash five times. Add 90 μl TMB substrate solution per well, cover with a plate sealer, and incubate for 15 min in the dark on the shaker at 37°C. Add 50 μl of stop solution to each well. Record the OD at 450 nm.

### Statistical Analyses

Statistical analysis was carried out by using SPSS (version 26.0, IBM, Armonk, NY, USA). The original baseline differences between the adult and pediatric groups were evaluated by using *t*-test for continuous variables and the chi-square test for categorical variables. A correlation analysis for the relationship between different serum biomarkers and age was performed. To further investigate the potential pathogenic mechanism differences between hemorrhagic MMD and ischemic MMD, we conducted subgroup analysis in the adult patients. Receiver operating characteristic (ROC) curve analysis was performed to evaluate the utility of a continuous biomarker. The cutoff point selection in the context of ROC curve analysis was according to the maximum of the Youden index. Furthermore, a conditional logistic regression analysis was performed to identify the independent risk factors of spontaneous hemorrhage in MMD.

## Results

### Patient Characteristics

The final cohort consisted of 84 patients diagnosed as having MMD (42 males and 42 females; 32.7 ± 17.3 years of age). The patient demographics are summarized in Table 1. Of these patients, 32 (32/84, 38.1%) experienced spontaneous hemorrhages, while 52 (52/84, 61.9%) presented with transient ischemic attacks (37/84, 44.0%) and prior cerebral infarctions (15/84, 17.9%). The Suzuki stage of the patients mostly was stage III (26/84, 31.0%) and stage IV (20/84, 23.8%). Patients experienced spontaneous hemorrhages present with a higher-grade Suzuki stage (*P* = 0.046).

**Table 1:** Baseline characteristics.

	**Adult**	**Pediatric**	***P***
	**(*n* = 59)**	**(*n* = 25)**	
Age, years	42.3 ± 10.2	9.84 ± 3.78	<0.0001
Female	29 (49.2)	13 (52.0)	0.811
BMI	24.3 ± 3.2	20.9 ± 5.2	0.002
Time of stroke events^a^			0.601
3–6 months	20 (48.8)	3 (50.0)	
>6 months	21 (51.2)	3 (50.0)	
mRS score at admission			0.890
0	6 (18.8)	5 (20.0)	
1–2	25 (78.1)	19 (76.0)	
3	1 (3.1)	1 (4.0)	
Medical history			
Hypertension	14 (23.7)	0 (0)	0.008
Diabetes mellitus	5 (8.5)	0 (0)	0.133
Hyperlipemia	5 (8.5)	0 (0)	0.133
Smoking	12 (20.3)	0 (0)	0.015
Suzuki stage			0.108
I	3 (5.1)	0 (0)	
II	5 (8.5)	6 (24.0)	
III	21 (35.6)	5 (20.0)	
IV	11 (18.6)	9 (36.0)	
V	11 (18.6)	3 (12.0)	
VI	8 (13.6)	2 (8.0)	
Posterior involvement	10 (16.9)	12 (48.0)	0.003
Unilateral lesion	6 (10.2)	3 (12.0)	0.804
rCBF↓	25 (42.4)	15 (60.0)	0.139

*Data are n (%) unless otherwise indicated. Mean values are given with SDs.*

*BMI, body mass index; mRS, modified Rankin Scale; rCBF, regional cerebral blood flow; rCBF↓ means decrease of rCBF.*

a *Forty-seven patients in the present study had stroke events, 32 patients had hemorrhagic stroke, and 15 patients had ischemic stroke*.

### Serum Level of MMP-9 Was Higher in Adult MMD Than in Pediatrics

**Figure 1:**
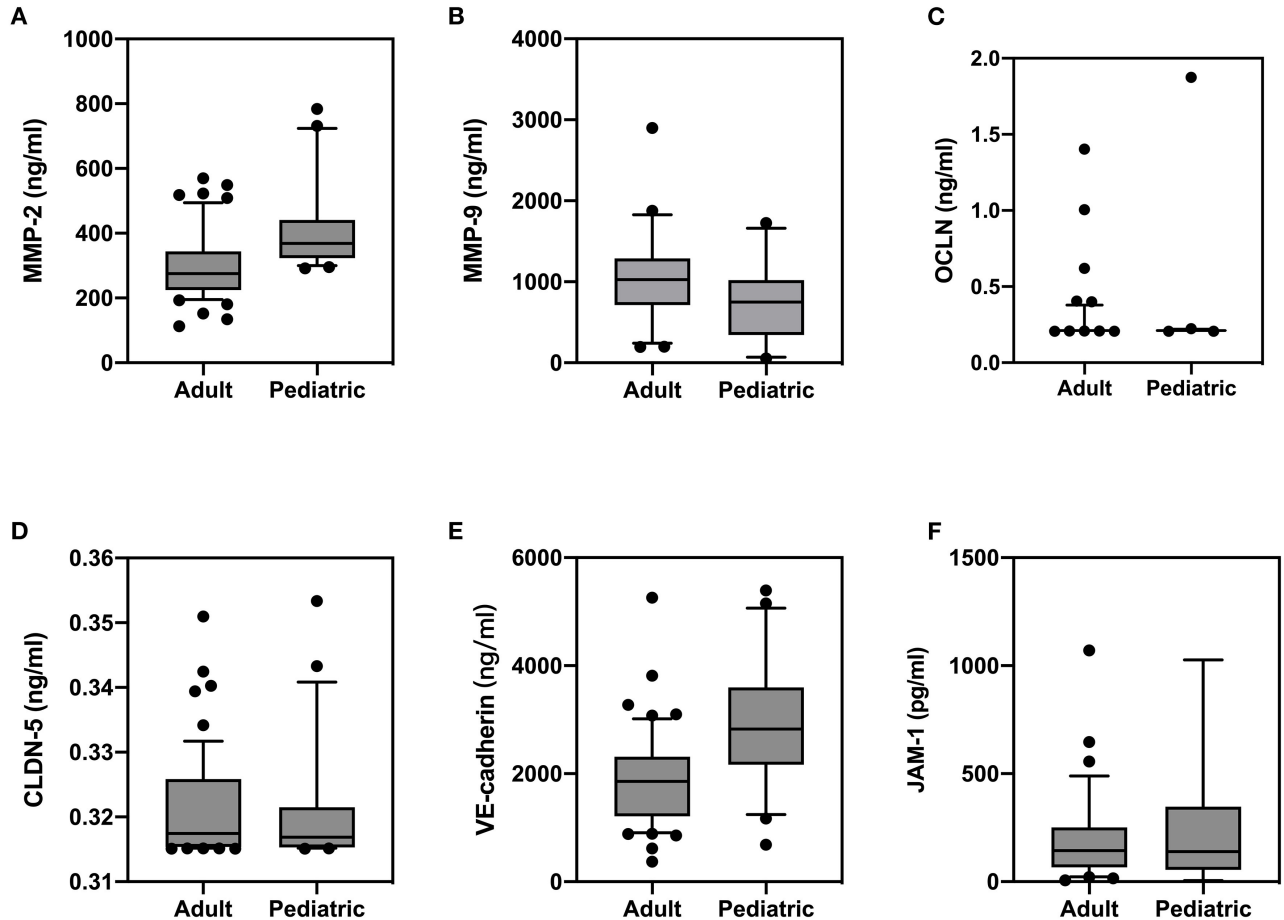
Comparison of serum levels of matrix metalloproteinase (MMP)-2 **(A)**, MMP-9 **(B)**, occludin (OCLN) **(C)**, claudin-5 (CLDN5) **(D)**, vascular endothelial (VE)-cadherin **(E)**, and junction-associated molecule (JAM)-1 **(F)** between adult and pediatric patients with and without spontaneous hemorrhages.

**Figure 2:**
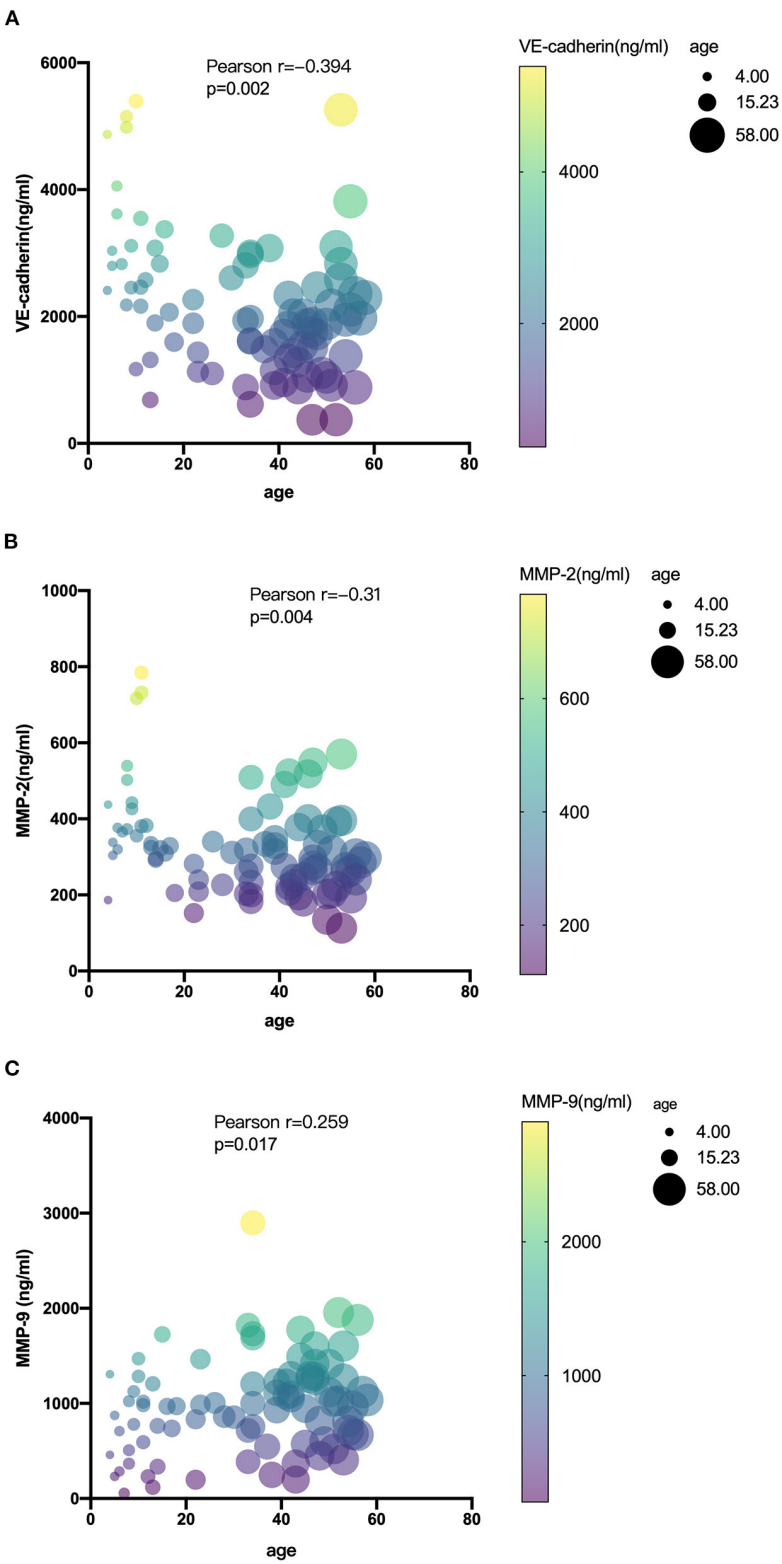
Correlations among serum levels of VE-cadherin **(A)**, MMP-2 **(B)**, and MMP-9 **(C)** with age in MMD.

The level of serum biomarkers tested by ELISA is summarized in Figure 1. Compared with pediatric patients, the serum level of MMP-9 was significantly elevated in adult patients (1,062 vs. 765 ng/ml; *P* = 0.012), while the serum level of MMP-2 was decreased (296 vs. 407 ng/ml; *P* < 0.0001) in adult patients. The serum level of VE-cadherin was also decreased significantly in adult patients compared with that in pediatric patients (1,847 vs. 2,961 ng/ml; *P* < 0.0001). There were no significant differences in the levels of OCLN, CLDN5, and JAM-1 between the two groups. A correlation analysis revealed that age exhibited a significant positive correlation with MMP-9 and a negative correlation with MMP-2 and VE-cadherin (Figure 2).

### The Serum Level of MMP-9 Was Significantly Upregulated in Hemorrhagic MMD Patients Than in Ischemic MMD Patients

**Figure 3:**
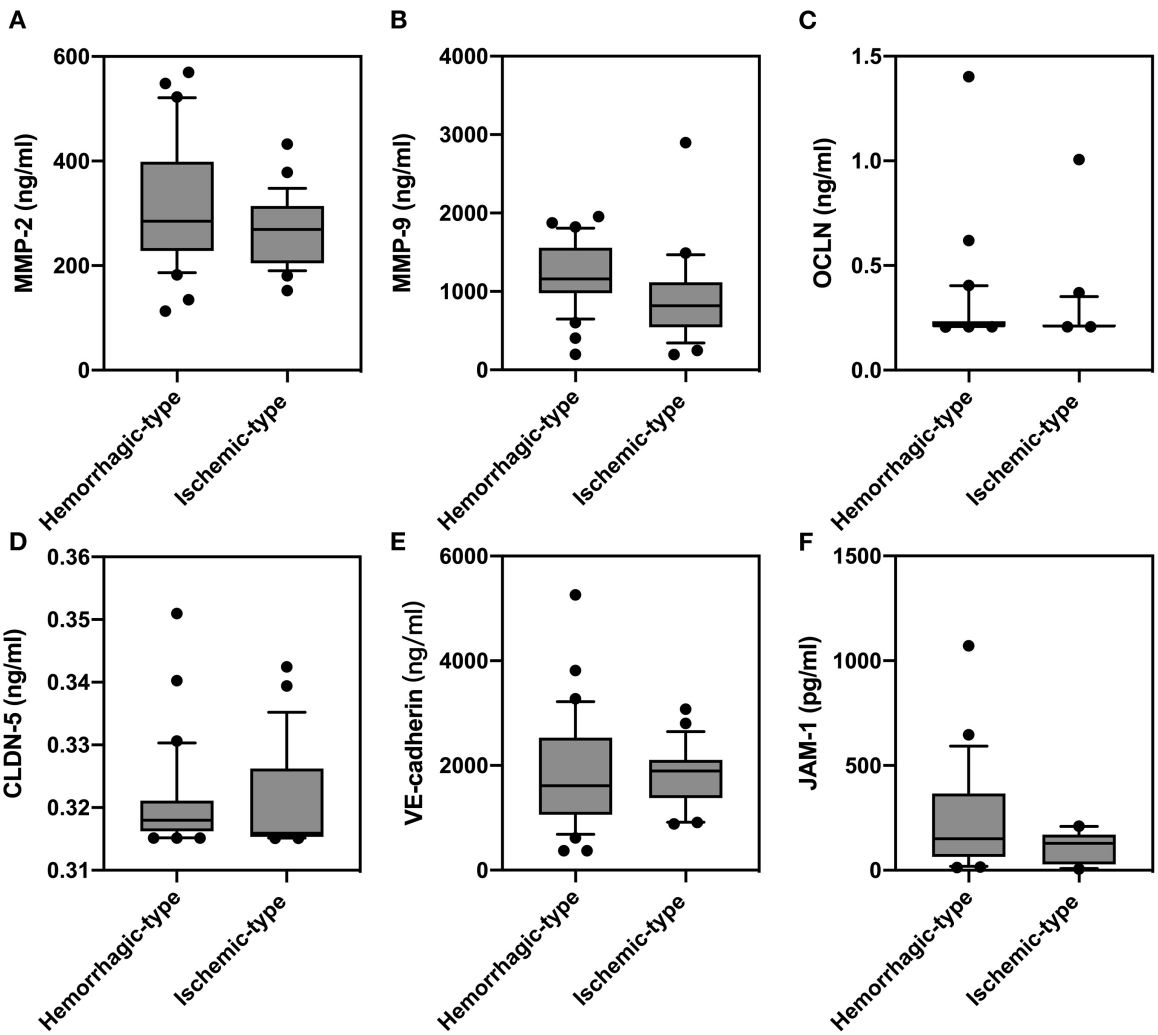
Comparison of serum levels of matrix metalloproteinase (MMP)-2 **(A)**, MMP-9 **(B)**, occludin (OCLN) **(C)**, claudin-5 (CLDN5) **(D)**, vascular endothelial (VE)-cadherin **(E)**, and junction-associated molecule (JAM)-1 **(F)** between adult patients with and without spontaneous hemorrhages.

**Figure 4:**
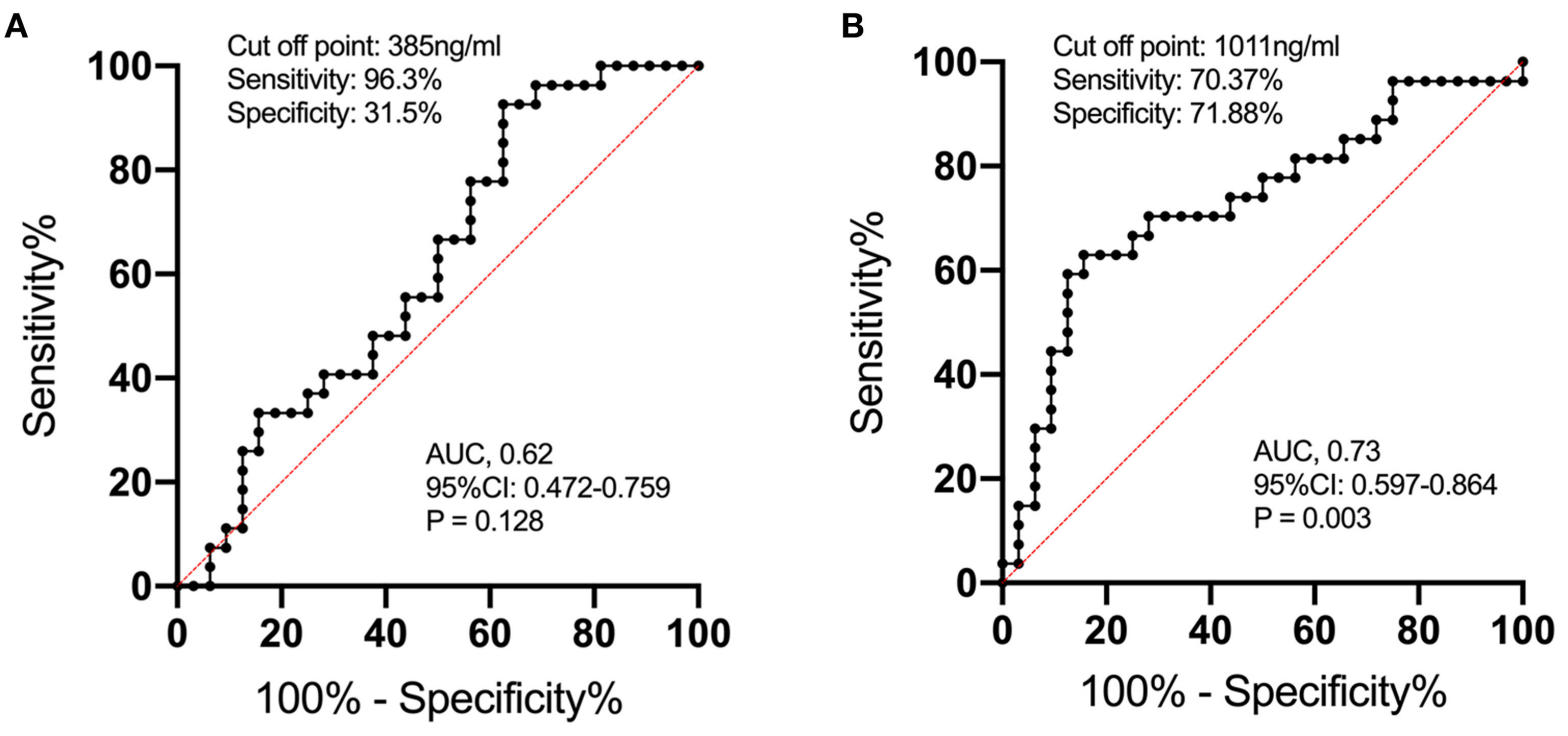
The ROC curve analysis of serum MMP-2 **(A)** and MMP-9 **(B)** concentrations for predicting the occurrence of spontaneous hemorrhage in MMD. The ROC curve identified that serum MMP-9 levels >1,011 ng/ml may predict the occurrence of spontaneous hemorrhage in adult patients with MMD with 70.37% sensitivity and 71.88% specificity [area under curve (AUC), 0.73; 95% CI 0.597–0.864; *P* = 0.003].

In order to study the expression of biomarkers among MMD subtypes, we compared the level of MMPs and tight junction proteins between adult ischemic and hemorrhagic MMD patients. The baseline characteristics of adult patients are presented in Table 2. Comparison of serum levels of various biomarkers between patients with and without spontaneous hemorrhages is presented in Figure 3. Compared with ischemic-type MMD patients, hemorrhagic-type MMD patients had significantly elevated serum level of MMP-9 and MMP-2 (1,186.13 vs. 917.74 ng/ml; *P* = 0.018 and 326.73 vs. 258.01 ng/ml; *P* = 0.05, respectively), while no significant differences were observed in the levels of OCLN, CLDN5, VE-cadherin, and JAM-1 between the two groups (Figure 3). Moreover, a ROC curve identified that a serum MMP-9 level >1,011 ng/ml was associated with spontaneous hemorrhage in adult MMD patients with 70.37% sensitivity and 71.88% specificity [area under curve (AUC), 0.73; 95% CI 0.597–0.864; *P* = 0.003] (Figure 4).

**Table 2:** Characteristics of study patients in the adult subgroup.

	**Hemorrhagic type**	**Ischemic type**	***P***
	**(*n* = 32)**	**(*n* = 27)**	
Age, years	43.8 ± 9.1	40.5 ± 11.2	0.218
Female	16 (50.0)	13 (48.1)	0.887
BMI	24.0 ± 3.0	24.7 ± 3.4	0.443
mRS score at admission			0.649
0	6 (18.8)	5 (18.5)	
1–2	25 (78.1)	22 (81.5)	
3	1 (3.1)	0 (0)	
Medical history			
Hypertension	9 (29.0)	5 (18.5)	0.351
Diabetes mellitus	2 (6.5)	3 (11.1)	0.656
Hyperlipemia	3 (9.7)	2 (7.4)	0.759
Smoking	5 (16.1)	7 (25.9)	0.358
Suzuki stage			0.242
I	2 (6.3)	1 (3.7)	
II	3 (9.4)	2 (7.4)	
III	9 (28.1)	12 (44.4)	
IV	4 (12.5)	7 (25.9)	
V	7 (21.9)	4 (14.8)	
VI	7 (21.9)	1 (3.7)	
Posterior involvement	5 (15.6)	5 (18.5)	0.768
Unilateral lesion	3 (9.4)	3 (11.1)	0.826
rCBF↓	15 (46.9)	10 (37.0)	0.446

*Data are n (%) unless otherwise indicated. Mean values are given with SDs.*

*BMI, body mass index; mRS, modified Rankin Scale; rCBF, regional cerebral blood flow; rCBF↓ means decrease of rCBF*.

### Analysis of Potential Spontaneous Hemorrhage Risk Factors for Adult Patients With MMD

After adjusting for potential covariables (Table 3), the logistic regression analyses showed that high-grade Suzuki stage (OR 4.565, 95% CI 1.028–20.280, *P* = 0.046) and serum MMP-9 level >1,011 ng/ml (OR 7.218, 95% CI 1.826–28.533, *P* = 0.005) were independent risk factors of spontaneous hemorrhages. Sex, age, current cigarette smoking, hypertension, diabetes mellitus, hyperlipemia, posterior circulation involvement, unilateral lesion, and decreased rCBF were not associated with any increased risk of spontaneous hemorrhages in the analysis (*P* > 0.05).

**Table 3:** Logistic regression analysis of predictors for spontaneous hemorrhages in adult patients with moyamoya disease.

**Covariate**	**Univariable**		**Multivariable**	
	**OR (95% CI)**	***P***	**OR (95% CI)**	***P***
Sex	0.929 (0.333–2.587)	0.887	2.037 (0.498–8.328)	0.322
Age	1.033 (0.981–1.089)	0.216	1.017 (0.955–1.083)	0.594
Smoking	0.529 (0.146–1.913)	0.332	0.287 (0.053–1.563)	0.149
Hypertension	1.722 (0.498–5.948)	0.390	1.059 (0.215–5.220)	0.944
Diabetes mellitus	0.533 (0.082–3.453)	0.510	0.482 (0.032–7.195)	0.597
Hyperlipemia	1.293 (0.200–8.369)	0.787	1.182 (0.116–12.009)	0.888
Suzuki stage >4	3.422 (1.035–11.318)	0.044	4.565 (1.028–20.280)	0.046
Posterior involvement	0.815 (0.209–3.179)	0.768	0.511 (0.088–2.980)	0.456
Unilateral lesion	0.828 (0.153–4.482)	0.826	0.987 (0.141–6.920)	0.989
rCBF↓	1.500 (0.527–4.265)	0.447	1.073 (0.240–4.805)	0.926
MMP-9 >1,011 ng/ml	6.069 (1.961–18.783)	0.002	7.218 (1.826–28.533)	0.005

*rCBF, regional cerebral blood flow; MMP-9, matrix metalloproteinase-9; rCBF↓ means decrease of rCBF*.

## Discussion

The pathophysiology of MMD has been limitedly investigated owing to the difficulty to harvest specimen and the lack of *in vitro* and *in vivo* models. It has been widely acknowledged that clinical manifestations and prognosis vary between MMD subtypes; however, the underlying mechanisms which lead to distinguished phenotypes remain poorly understood. MMP-9, also known as gelatinase B, which plays an important role in extracellular matrix degradation and endothelial junctions breakdown, is recently found involved in MMD (17). Increased expression of MMP-9 in the serum and in vessel specimen of MMD patients has been reported (13); however, whether MMP-9 is associated with clinical presentations of MMD has not been investigated. In this study, we investigate the expression of MMPs and BBB biomarkers between different MMD subtypes, hoping to further elucidate the role of MMP-9 in MMD and find a pathophysiologic basis for MMD phenotypes.

Our results showed that the serum level of MMP-9 was significantly higher in adults than that in pediatric MMD patients. Age is significantly correlated with the serum level of MMP-9 and VE-cadherin (Figure 2). The level of MMP-9 increases and VE-cadherin decreases as the age of patients rises, indicating later MMD onsets, higher gelatinase activity, and poorer BBB function the patients might have, which might be related with hemorrhagic stroke presentations. However, it should be noted that the significant difference of hypertension, BMI, and smoking in the two groups might be responsible for the difference in MMP levels between adults and children. Propensity score matching can be used to reduce imbalances in the baseline characteristics between adults and children. Due to the limited sample size of the present study, the logistic regression model which determines the propensity score might unreasonable. Thus, further study should include a larger sample size to adjust for these confounding factors, so as to obtain more accurate results.

Given the fact that almost all pediatric patients present ischemic strokes, we further investigate MMP-9 in adult MMD subgroups and results showed, similarly, that hemorrhagic-type MMD patients had a higher level of MMP-9 than ischemic-type MMD patients. MMP-9 degrades endothelial basal lamina and tight junction proteins by digesting type IV collagen, of which its excessive activation leads to endothelial instability and eventually causes BBB breakdown (18). Correspondingly, we also found that the serum level of VE-cadherin was significantly lower in hemorrhagic-type MMD patients than in ischemic-type patients. Previous studies pointed out that hemorrhagic-type MMD might be related with BBB dysfunction, as more microbleeds were noticed on MRI in hemorrhagic-type than in ischemic-type MMD (19). In accordance, our findings also indicated that BBB permeability is higher in hemorrhagic-type MMD patients. Together, it might be postulated that MMP-9 activity and BBB disorder are associated with the risk of hemorrhage in MMD patients.

Given this, the serum level of MMP-9 might serve as a biomarker for predicting hemorrhage in MMD patients. We performed a ROC analysis and results showed that a serum MMP-9 level >1,011 ng/ml was associated with spontaneous hemorrhage in adult MMD patients with 70.37% sensitivity and 71.88% specificity (AUC 0.73, 95% CI 0.597–0.864, *P* = 0.003) (Figure 4). To rule out confounding factors, we further conducted multivariate analysis including age and other potential risk factors (smoking, hypertension, vasculature characteristics, etc.), and results showed that serum MMP-9 level >1,011 ng/ml is an independent risk factor of MMD hemorrhagic strokes (*P* = 0.005, Table 3). This finding might be very useful for the identification of hemorrhagic MMD and further stroke predictions. Future studies are needed to verify the predictive value of MMP-9 for hemorrhagic strokes in MMD.

MMP-9 also participates in angiogenesis with dual function (20). At the early stage of angiogenesis, regulated by vascular endothelial growth factor, MMP-9 expression increases, leading to endothelial junction breakdown, releasing hidden VEGF binding sites and promoting pruning (21). At the late stage, overactivation of MMP-9 exhibits anti-angiogenesis effect and causes destruction of newly formed vessels also by destabilizing cellular junctions and upregulating angiostatin and endostatin (22, 23). Our previous study showed that after indirect bypass surgery, hemorrhagic-type MMD formed limited angiogenesis compared with ischemic-type MMD, most of which were very well-revascularized, though the pathophysiological cause was unclear (7). Considering the current study, the high activity of MMP-9 might be a reason for poor angiogenesis in hemorrhagic MMD and could be a potential pharmaceutical target to promote the surgical effect for MMD. However, due to the retrospective nature of this study, the patients included who underwent indirect bypass were insufficient to render a valid result. The role of MMP-9 in surgical effect in MMD will definitely be investigated in further studies.

On the other hand, MMP inhibition is mediated by tissue inhibitors of metalloproteinases (TIMPs) that coexist with MMPs. TIMPs inactivate the activity of MMPs to prevent excessive tissue degradation and injury. However, it should be noted that this study is a preliminary prospective observational study, and further study will be conducted under the results of this study. Thus, the TIMPs have not been evaluated in the present study. We will further explore whether the TIMPs and MMP/TIMP ratio are associated with MMD subtypes in follow-up studies.

There are study limitations that need to be addressed for the accurate interpretation of our data. First, the serum levels of MMP-9 and BBB-related proteins were compared between MMD subgroups, but not with healthy control. However, previous studies have already compared the expression pattern of aforementioned biomarkers in MMD patients and healthy controls. Second, this is a preliminary investigation of MMP-9 and BBB function between MMD subtypes. The predictive value for hemorrhage prediction and pharmaceutical value for improving the surgical effect of MMP-9 needs to be validated in future studies. Third, further studies are needed to explore the exact role of MMP-9 function and BBB impairment in MMD pathophysiology. Lastly, due to the single cohort analysis nature of the study, the versatility of the results is limited, and further validation in another cohort is necessary.

## Conclusions

Hemorrhagic-type MMD patients had a higher serum level of MMP-9 and BBB permeability compared with ischemic-type MMD patients. Adult MMD patients had a higher serum level of MMP-9 and BBB permeability compared with pediatric patients. MMP-9 might serve as a biomarker for hemorrhage prediction in MMD. Serum MMP-9 level >1,011 ng/ml is an independent risk factor of MMD hemorrhagic strokes.

## Data Availability Statement

The raw data supporting the conclusions of this article will be made available by the authors, without undue reservation.

## Ethics Statement

The studies involving human participants were reviewed and approved by Beijing Tiantan Hospital Research Ethics Committee. Written informed consent to participate in this study was provided by the participants' legal guardian/next of kin.

## Author Contributions

JL and JW designed the study, wrote the manuscript, researched the data, and contributed to the discussion. ZL researched the data and edited the manuscript. GS, RW, and JZ contributed to the discussion and edited the manuscript. YaZ and YuZ reviewed and edited the manuscript and contributed to the discussion. All authors contributed to the article and approved the submitted version.

## Conflict of Interest

The authors declare that the research was conducted in the absence of any commercial or financial relationships that could be construed as a potential conflict of interest.

## Publisher's Note

All claims expressed in this article are solely those of the authors and do not necessarily represent those of their affiliated organizations, or those of the publisher, the editors and the reviewers. Any product that may be evaluated in this article, or claim that may be made by its manufacturer, is not guaranteed or endorsed by the publisher.
