# Supplementary material for: MMP-9 as a Biomarker for Predicting Hemorrhagic Strokes in Moyamoya Disease
Source: Front Neurol. 2021 Aug 31;12:721118. doi: 10.3389/fneur.2021.721118 (PMC8438170; doi:10.3389/fneur.2021.721118)
Supplement: Supplementary file 1 [file Data_Sheet_1.PDF]

To verify the accuracy of ROC analysis, a hold-out method of model validation was used, whereby the data was randomly split in half into training and validation data sets (Table 1). In the training data set, the ROC curve identified that that serum MMP-9 levels > 1002 ng/ml were associated with spontaneous hemorrhage in adult MMD. (Figure 1) After adjusting for potential covariables (Table 2), the logistic regression analyses showed that serum MMP-9 level > 1002 ng/ml (OR 34.514, 95% CI 1.886-631.705, p = 0.017) were independently risk factors of spontaneous hemorrhages in the validation data set.

Table 1. Characteristics of study patients in the training and validation data sets

| | Training data set (n=29) | Validation data set (n=30) |
|---|---|---|
| Age, yrs | 45.2 ± 10.2 | 39.4 ± 9.1 |
| Female | 11 | 18 |
| BMI | 24.0 ± 2.7 | 24.6 ± 3.4 |
| mRS score at admission | | |
| 0 | 2 | 9 |
| 1-2 | 27 | 20 |
| 3 | 0 | 1 |
| Medical history | | |
| Hypertension | 8 | 6 |
| Diabetes mellitus | 3 | 2 |
| Hyperlipemia | 1 | 4 |
| Smoking | 9 | 3 |
| Suzuki stage | | |
| I | 2 | 1 |
| II | 4 | 1 |
| III | 12 | 9 |
| IV | 6 | 5 |
| V | 2 | 9 |
| VI | 3 | 5 |
| Posterior involvement | 3 | 7 |

| | | |
|---|---|---|
| Unilateral lesion | 3 | 3 |
| rCBF↓ | 15 | 10 |

BMI = body mass index; mRS = modified Rankin Scale ; rCBF = regional cerebral blood flow

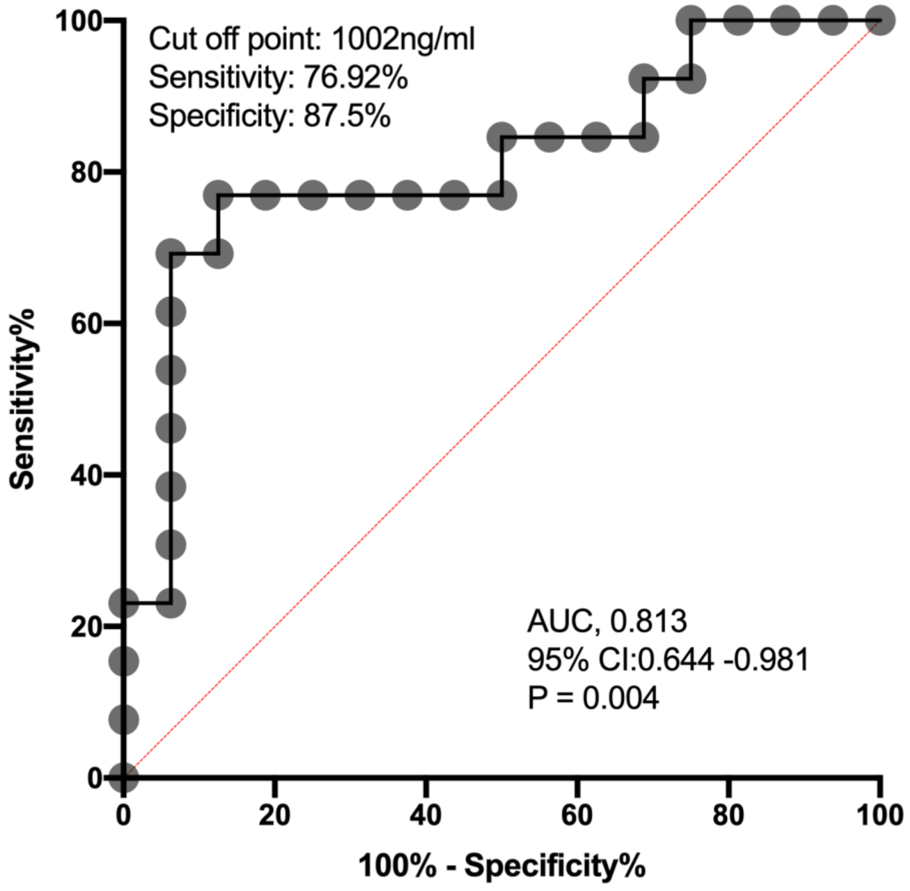


Figure 1. The ROC curve analysis of serum MMP-9 (B) concentration for predicting the occurrence of spontaneous hemorrhage in MMD in the training data set.

Table 2. Univariable and multivariable analysis of adult moyamoya disease presentation with hemorrhage in validation data set (n=30)

| | Univariable | | Multivariable | |
|---|---|---|---|---|
| Covariate | OR (95%CI) | p Value | OR (95%CI) | p Value |
| Sex | 0.455 (0.103-20.13) | 0.299 | 0.568 (0.065-4.991) | 0.610 |
| Age | 1.017 (0.940-1.101) | 0.671 | 1.009 (0.883-1.152) | 0.899 |
| Hypertension | 0.846 (0.141-5.070) | 0.855 | 0.453 (0.040-5.139) | 0.523 |
| Hyperlipemia | 0.857 (0.104-7.043) | 0.886 | 4.648 (0.163-132.612) | 0.369 |
| Suzuki stage > 4 | 4.167 (0.894-19.419) | 0.069 | 1.565 (0.180-13.592) | 0.685 |
| Posterior involvement | 0.577 (0.104-3.186) | 0.528 | 0.284 (0.022-3.602) | 0.332 |
| Unilateral lesion | 0.400 (0.032-4.960) | 0.476 | 0.438 (0.010-19.710) | 0.671 |
| rCBF↓ | 2.852 (0.568-14.326) | 0.203 | 0.941 (0.055 16.022) | 0.967 |
| MMP-9 > 1002ng/ml | 17.500 (2.667-114.846) | 0.003 | 34.514 (1.886-631.705) | 0.017 |

Table 3. Time stroke event occurred in each patient.

| Patients No. | Sex | Age group/y | Stroke Type | Time of Stroke/month | mRS Score |
|---|---|---|---|---|---|
| 1 | Female | 41-50 | Hemorrhagic | 3 | 1 |
| 2 | Female | 21-30 | Ischemic | 3 | 1 |
| 3 | Male | 41-50 | Hemorrhagic | 16 | 0 |
| 4 | Male | 51-60 | Hemorrhagic | 5 | 1 |
| 5 | Female | 11-20 | Ischemic | 7 | 1 |
| 6 | Female | 41-50 | Ischemic | 3 | 1 |
| 7 | Female | 41-50 | Hemorrhagic | 14 | 1 |
| 8 | Female | 31-40 | Hemorrhagic | 18 | 0 |
| 9 | Female | 31-40 | Ischemic | 12 | 0 |
| 10 | Female | 31-40 | Hemorrhagic | 48 | 3 |
| 11 | Female | 31-40 | Hemorrhagic | 6 | 1 |
| 12 | Female | 41-50 | Ischemic | 16 | 1 |
| 13 | Female | 41-50 | Hemorrhagic | 3 | 1 |
| 14 | Male | 1-10 | Ischemic | 3 | 1 |
| 15 | Male | 41-50 | Ischemic | 12 | 1 |
| 16 | Male | 41-50 | Ischemic | 3 | 1 |
| 17 | Female | 41-50 | Hemorrhagic | 12 | 0 |
| 18 | Male | 51-60 | Ischemic | 48 | 0 |
| 19 | Male | 1-10 | Ischemic | 96 | 2 |
| 20 | Female | 11-20 | Ischemic | 3 | 2 |
| 21 | Female | 11-20 | Hemorrhagic | 8 | 0 |
| 22 | Female | 41-50 | Hemorrhagic | 11 | 0 |
| 23 | Male | 41-50 | Hemorrhagic | 12 | 0 |
| 24 | Male | 21-30 | Hemorrhagic | 3 | 1 |
| 25 | Male | 21-30 | Ischemic | 3 | 2 |
| 26 | Male | 41-50 | Ischemic | 3 | 1 |
| 27 | Female | 41-50 | Hemorrhagic | 24 | 1 |
| 28 | Male | 31-40 | Hemorrhagic | 3 | 1 |
| 29 | Female | 41-50 | Hemorrhagic | 22 | 1 |
| 30 | Male | 51-60 | Hemorrhagic | 6 | 1 |

| | | | | | |
|---|---|---|---|---|---|
| 31 | Female | 51-60 | Hemorrhagic | 4 | 1 |
| 32 | Female | 51-60 | Ischemic | 12 | 1 |
| 33 | Female | 41-50 | Hemorrhagic | 3 | 1 |
| 34 | Female | 31-40 | Hemorrhagic | 5 | 1 |
| 35 | Female | 41-50 | Hemorrhagic | 6 | 1 |
| 36 | Male | 41-50 | Hemorrhagic | 3 | 1 |
| 37 | Male | 51-60 | Hemorrhagic | 6 | 1 |
| 38 | Male | 51-60 | Hemorrhagic | 4 | 1 |
| 39 | Female | 41-50 | Hemorrhagic | 19 | 1 |
| 40 | Male | 31-40 | Hemorrhagic | 4 | 1 |
| 41 | Female | 1-10 | Ischemic | 3 | 3 |
| 42 | Male | 31-40 | Hemorrhagic | 6 | 1 |
| 43 | Male | 51-60 | Hemorrhagic | 3 | 2 |
| 44 | Male | 51-60 | Hemorrhagic | 6 | 1 |
| 45 | Male | 51-60 | Hemorrhagic | 4 | 1 |
| 46 | Male | 41-50 | Hemorrhagic | 3 | 2 |
| 47 | Male | 51-60 | Hemorrhagic | 3 | 1 |
